# Drainage during Condensation on Microgrooved Biphilic Surfaces

**DOI:** 10.1021/acs.langmuir.3c02433

**Published:** 2023-12-29

**Authors:** Daniel Fotachov, Raphael Raab, Hans-Jörg Bart, Egbert Oesterschulze

**Affiliations:** † Department of Physics, Physics and Technology of Nanostructures, Rhineland-Palatinate Technical University of Kaiserslautern-Landau, Kaiserslautern 67663, Germany; ‡ Fluidverfahrenstechnik, Rhineland-Palatinate Technical University of Kaiserslautern-Landau, Kaiserslautern 67663, Germany

## Abstract

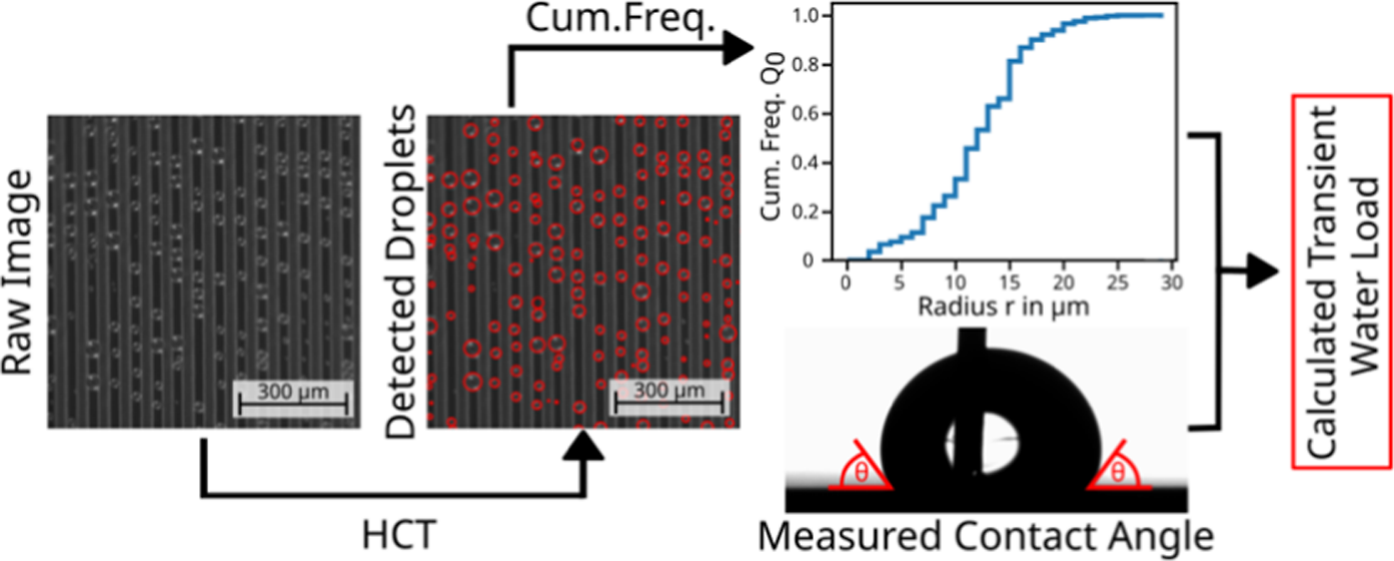

In this work, we investigate and compare the condensation behavior of hydrophilic, hydrophobic, and biphilic microgrooved silicon samples etched by reactive ion etching. The microgrooves were 25 mm long and 17–19 μm deep with different topologies depending on the etching process. Anisotropically etched samples had 30 μm wide rectangular microgrooves and silicon ridges between them. They were either left hydrophilic or covered with a hydrophobic fluorocarbon or photoresist layer. Anisotropically etched samples consisted of 48 μm wide semicircular shaped microgrooves, 12 μm wide silicon ridges between them, and a 30 μm wide photoresist stripe centered on the ridges. The lateral dimensions were chosen to be much smaller than the capillary length of water to support drainage of droplets by coalescence rather than droplet sliding. Furthermore, to achieve a low thermal resistance of the periodic surface structure consisting of water-filled grooves and silicon ridges, the trench depth was also kept small. The dripped-off total amount of condensate (AoC) was measured for each sample for 12 h under the same boundary conditions (chamber temperature 30 °C, cooling temperature 6 °C, and relative humidity 60%). The maximum increase in AoC of 15.9% (9.6%) against the hydrophilic (hydrophobic) reference sample was obtained for the biphilic samples. In order to elucidate their unique condensation behavior, *in situ* optical imaging was performed at normal incidence. It shows that the drainage of droplets from the stripe’s surface into the microgrooves as well as occasional droplet sliding events are the dominant processes to clear the surface. To rationalize this behavior, the Hough Circle Transform algorithm was implemented for image processing to receive additional information about the transient droplet size and number distribution. Postprocessing of these data allows calculation of the transient water load on the stripe’s surface, which shows an oscillatory behavior not previously reported in the literature.

## Introduction

Condensation is an important technical and energy consuming process in industrial applications such as power generation, water desalination, thermal management, freezing, or air conditioning. The condensing plate as key component has been the focus of recent research.^1,2^ It should combine an excellent thermal conductivity for energy throughput with an effective condensing surface. This demands the effective removal of the condensate, in particular water, with its low thermal diffusivity hindering thermal energy throughput. In principle, two types of condensation phenomena are distinguished: filmwise condensation (FWC) and dropwise condensation (DWC).^3^ FWC refers to the formation of a condensation layer of growing thickness covering the entire surface, thereby thermally isolating the surface from the vapor and reducing the overall heat transfer owing to the low thermal diffusivity of water.^3−5^ In DWC, however, individual droplets condense on the surface, resulting in up to an order of magnitude higher heat transfer.^6−9^

Another approach to increasing heat transfer is to use a hybrid structure of hydrophilic and hydrophobic areas, called biphilic surfaces.^10^ Not only droplet removal is increased by the hydrophobic areas but also droplet nucleation is improved by the hydrophilic areas.^11,12^ Daniel et al. (2001) have shown that droplets tend to move from areas with low wettability toward high wettability, resulting in additional droplet removal and mass transfer.^13^ Chen et al. (2011) fabricated hierarchical nanograss decorated micropyramids with local hydrophilic nucleation sites on a superhydrophobic surface, resulting in a 65% increase in droplet density.^14^ By using alternating stripes of hydrophilic and hydrophobic material, Peng et al. (2014) showed, that it is possible to limit the maximum diameter of condensing droplets, before they are removed, thereby creating new nucleation sites for the condensate.^15^ By optimizing the ratio of hydrophilic to hydrophobic area, as well as the area of each stripe, it may be possible to increase the heat transfer.^16−18^ The incorporation of a third dimension in the form of a microstructure or nanostructure may be useful to further improve the wetting behavior of biphilic surfaces.^19^

Depending on parameters such as the depth of the structure, the area ratio of the hydrophilic to hydrophobic surfaces and their size, droplets can be drained, creating a thin film with approximately the same depth as the structure, resulting in a thin liquid layer inside the grooves with only weak insulating properties.^20^ Lo et al. have demonstrated, that by using a 3D hybrid surface, consisting of hydrophilic channels and nanostructured superhydrophobic plateaus, the heat transfer coefficient and heat flux can be increased by 84% compared to superhydrophobic silicon nanowires.^21^ Here, the ratio of the hydrophobic region and the hydrophilic region was always 1.5, with a width of the hydrophilic microchannels of 300, 600, and 1300 μm and a depth of 50 μm. The superior heat transfer was attributed to the large wettability gradient on the surface, the thin film thickness inside the grooves, the absence of liquid bridges that cover several grooves, and the fast departure of droplets.

Here, we present results on the water condensation behavior of microgrooved silicon samples that are partially coated to provide both a structural and a wetting gradient. However, we have chosen the dimensions (width of grooves < 50 μm, groove spacing *ca.* 30 μm) well below the capillary length of water so that drainage of surface droplets by coalescence with water in the grooves is expected to become the dominant surface clearing effect for the stripes compared to droplet sliding events. Since drainage depends on the amount and size distribution of droplets on the stripe’s surface, we have implemented optical *in situ* imaging normal to the surface during condensation. We will show that by postprocessing of these images with an adapted Hough Circle Transform (HCT) algorithm, it is possible to evaluate both the number and the size distribution of droplets. By further assuming that each droplet forms a spherical cap on the surface and taking the advancing contact angle measured on a flat sample surface into account, we can calculate the transient amount of water on the stripe’s surface. This is discussed, in particular, for biphilic surfaces. Here, we focus on the unique oscillation behavior observed after droplet sliding, which has not been reported in the literature.

## Experimental Section

### Fabrication and Handling of Samples

**Figure 1:**
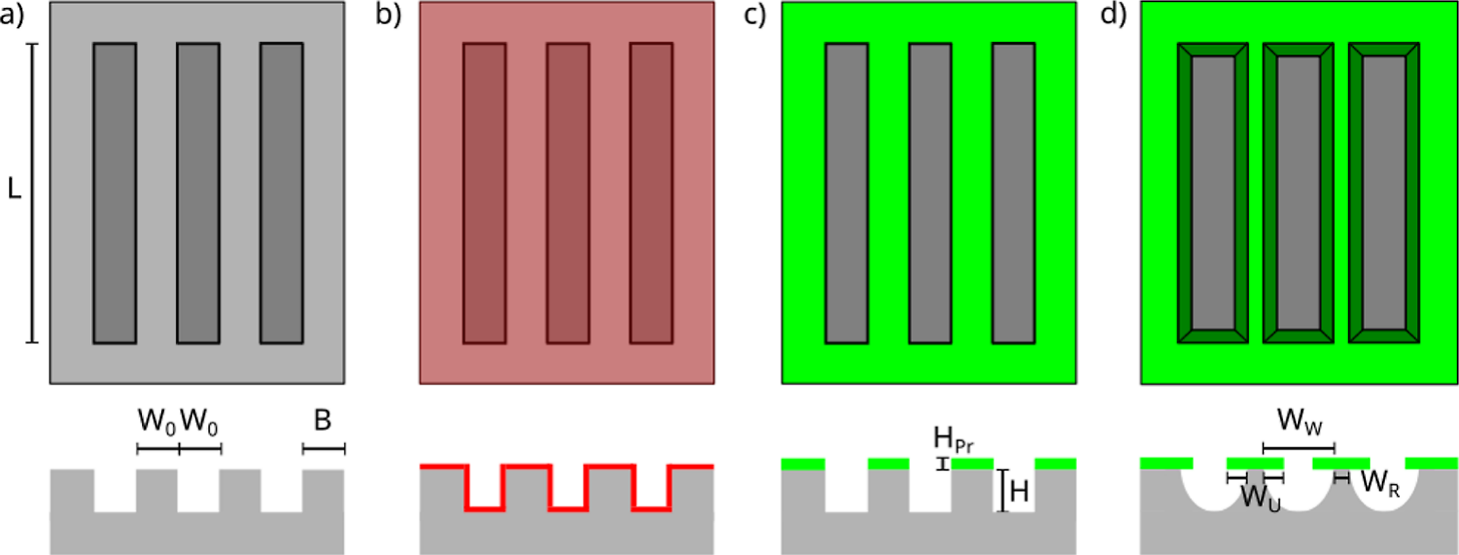
Schemes of the four microgrooved samples showing both top view and profile: (a) MSi-A, (b) F-MSi-A, (c) PrMSi-A, and (d) PrMSi-I. The light gray areas correspond to the top Si surface, and the dark gray areas to the bottom of the plasma etched silicon. The red color represents the hydrophobic fluorocarbon layer. The light green color indicates the photoresist layer left on the Si surface, while the dark green color stands for the underetched photoresist. Coating of the MSi-A sample in (a) with a fluorocarbon layer results in the F-MSi-A sample shown in (b).

Samples were processed from (100) oriented silicon wafers (4 in. diameter, double side polished). Six different sample configurations were considered with microgrooved (index M) or flat (no index) surfaces each with dimensions of 30 × 30 mm^2^ and different surface wetting properties. A flat unprocessed silicon sample (Si) with intrinsic hydrophilic wetting properties and a silicon sample (F–Si) plasma coated with an approximately 300 nm thin hydrophobic fluorocarbon layer (index F) serve as reference samples. In the case of the remaining four microgrooved samples, UV laser lithography was used to transfer the periodic stripe structure (stripe and groove width *W*_0_ = 30 μm each) into a 1.4 μm thin photoresist layer (AZ 1512 HS) spin-coated onto the wafer surface. Inductively coupled reactive ion etching (Oxford, PlasmaPro100 Cobra) was performed with two different sets of parameters to obtain microgrooved silicon (MSi) surfaces with anisotropic (index A) or isotropic (index I) etch profiles. Leaving the photoresist (index Pr) on the top surface after plasma etching (Figure 1c,d) resulted in biphilic samples (PrMSi-A and PrMSi-I) with the hydrophobic photoresist coating on top of the hydrophilic silicon ridges and also hydrophilic grooves. The removal of the photoresist layer results in a completely hydrophilic microgrooved silicon surface (MSi-A, Figure 1a). However, if the latter is homogeneously coated with a *ca.* 300 nm thin fluorocarbon layer, the sample surface becomes completely hydrophobic (F-MSi-A, Figure 1b).

After development, the rectangular openings in the photoresist layer had a width of *W*_0_ = 30 μm, a height of 1.4 μm, and a length of *L* = 25 mm (Figure 1). The reactive ion etching of silicon resulted in a reduction of the photoresist thickness to about *H*_Pr_ = 1 μm. The groove depth was *H* = 17 μm for the PrMSi-I and MSi-A samples and 19 μm for the PrMSi-A samples. The profile of the anisotropically etched surface resembles almost periodic rectangular grooves with a width of 30 μm and a mutual distance of 60 μm (Figure 1a). The isotropically etched surface of the PrMSi-I samples shows a semicircular etch profile with a maximum diameter *W*_W_ = 48 μm (Figure 1d). This results in 30 μm wide photoresist stripes centered on top of an only *W*_R_ = 12 μm wide silicon ridge with the same mutual distance of 60 μm between the ridges. The photoresist layer overhangs the ridges on each side by *W*_U_ = 9 μm.

### Experimental Setup and Analyzing Methods

A sample chiller (UEPT-KIT3-Pt100) consisting of a fan (UEPK-Lu-12-60), a Peltier cooling element (UEPT-140-127-040C200S), and an aluminum block serving as the sample mount was installed on a goniometer whose surface normal can be tilted up to 90°. This setup was installed in an environmental chamber (Memmert HPP 260) to provide stable condensation conditions (chamber temperature 30 °C, relative humidity 60%). The chamber log files listed maximum fluctuations of 0.5% for humidity and 0.5 °C for temperature. Measurements were conducted for 12 h. The overall sample dimensions of 30 × 30 mm^2^ was chosen to match the size of the aluminum cooling block. The microgrooved samples were designed with a flat border area of width *B* = 2.5 mm on all sides, reducing the structured surface to 25 × 25 mm^2^. This was necessary because the samples were attached to the cooling block with self-adhesive thermally well conducting tape (RS Pro Heat conduction pad, 4.5 W/mK). Applying pressure only to the border area with an adapted 3D printed mechanical stamp has been shown to improve both the mechanical and thermal contact of the sample to the cooling block substantially while leaving the microstructures undamaged.

The grooves on the samples were oriented vertically during the condensation to get less pinning of droplets sliding down the structured surface.^22^ An electronic scale (Kern EWJ 300-3, listed reproducibility: ±0.005 *g*) with a Petri dish on top was used to collect and measure *in situ* the total amount of condensate (AoC) dripping from the surface. A camera (ASI 1600 MM Mono, number of pixels: 4656 × 3520, maximum field of view: 2.18 × 1.65 mm^2^) was installed normal to the sample surface to image the condensation on the microstructure of the samples irrespective of the angle of rotation (NV imaging mode). A magnifying lens (10×) and a ring LED were installed on the camera (exposure time 0.5 s) to resolve growing droplets as small as 2 μm diameter both inside and outside the grooves.

The droplet radius distribution for the PrMSi-I surface and the PrMSi-A surface was determined by using the HCT algorithm for image processing. Details of how exactly the HCT was implemented can be found in the Supporting Information (Section S3). The application of the HCT allowed us to determine the radius and number of droplets that were on top of the hydrophobic stripes while the microgrooves were completely flooded. Droplets smaller than 1 μm in radius could not be detected owing to the limited resolution of the optical setup. By manually examining particular frames with a local minimum (maximum) of the transient water load, the radius of 99% (94%) of all droplets on the surface were determined successfully. With the given droplet radius distribution and measured advancing contact angle (Supporting Information, Section S1), the volume of water on the surface was calculated, assuming each droplet as a spherical segment. For the minimum water load, almost all droplets larger than 1 μm in radius could be detected. For a conservative estimation of the error determining the water amount on the surface, we assume that each unsuccessfully detected droplet has the maximum possible diameter of 40 μm. Then, the range of the error is 6.5% for the maximum and 2.1% for the minimum water load on top surface of the stripes.

A second camera (IDS UI-336xCP-M, number of pixels: 2048 × 1088) was installed to observe the condensation process on a macroscopic scale over the entire sample. It was installed under grazing incidence of about 5° to the surface (SV imaging mode) with a field of view of 30 × 8 mm^2^. To increase the exposure time, while keeping the frame rate constant, the sensitive camera area was cropped to 1072 × 300 pixels (Supporting Information, Section S2).

All measurements were performed with the goniometer tilted at 90°, i.e., with the surface normal in horizontal position, establishing a relative humidity of 60% at a chamber temperature of 30 °C and a cooling aluminum block temperature of 6 °C. This corresponds to a supersaturation value of 2.71. The imaging of the condensation process on the sample surface was carried out in a first period in both the SV and NV configurations for 45 min to study the condensation in its early stages. This was followed by another imaging cycle after 11.75 h for 12 and 30 min in NV and SV mode, respectively.

## Results and Discussion

### Results

The AoC collected for the different samples over a 12 h period (Supporting Information, Section S4) are summarized in Table 1. They were recorded under the boundary conditions mentioned in the experimental setup. In the following, the condensation behavior of each sample type is discussed with reference to that of the flat hydrophilic silicon sample (Si).

**Table 1:** AoC for Each Sample Accumulated Over a 12 h Period and Their Relative Difference with Respect to the AoC_Si_ of the Flat Silicon (Si) Surface

sample	AoC in g	(AoC – AoC_Si_)/AoC_Si_ in %
Si	18.62	reference
F–Si	19.70	+5.8
MSi-A	18.95	+1.8
F-MSi-A	19.51	+4.8
PrMSi-I	20.99	+12.7
PrMSi-A	21.59	+15.9

As a matter of fact, all samples collected more water than the flat Si reference sample. Condensation on the flat F–Si sample was based on DWC and showed an increase in AoC of 5.8% which is in good agreement with results reported for similar samples in the literature.^5^ The hydrophilic microgrooved silicon sample (MSi-A) shows only a minor increase in AoC compared with the flat Si surface. In the case of the F-MSi-A sample, the hydrophobic coating of the microgrooved surface gives almost the same amount of AoC as for the flat F–Si surface. While the MSi-A sample shows a mixture of DWC and FWC, the Si, F–Si, and F-MSi-A samples are restricted to DWC only.

In contrast to the samples discussed so far with their homogeneous hydrophobic or hydrophilic surface, we now turn to the samples with a biphilic surface. In the case of the PrMSi-I and PrMSi-A samples, we observed the largest increases in AoC of 12.7 and 15.9%, respectively. Both of these samples feature hydrophobic photoresist stripes on top of the silicon ridges separated by hydrophilic silicon microgrooves. During the experiments, no degeneration of the hydrophobic coating was observed. Although the PrMSi-A sample has 2 μm deeper microgrooves than the PrMSi-I sample and therefore a decreased vertical heat flux once filled with condensate, its AoC collected is still higher. However, in the case of the PrMSi-I sample, the isotropic underetching of the photoresist stripes by *W*_u_ = 9 μm (Figure 1c) from each side during plasma preparation results in silicon ridges of much smaller width *W*_0_ – 2*W*_u_ = 12 μm compared to their original size of *W*_0_ = 30 μm (Figure 1c), leading to an increased volume of water-filled microgrooves. The much lower ratio of the width of a single silicon ridge to that of a water-filled groove of 1:4 for the PrMSi-I sample compared to 1:1 for the PrMSi-A sample and the corresponding increase in water volume result in a lower total heat flux through the water-filled surface structure of the PrMSi-I sample. This may explain its reduced condensation rate compared to that of the PrMSi-A sample.

To investigate this phenomenon in more detail, we optically monitored the sample surface during condensation with two cameras. Imaging revealed that all surfaces initially condensed in DWC mode, revealing circular droplets. In addition, droplet sliding events resulted in satellites remaining on the swept surface, which act as condensation sites. Irrespective of the sample type, complete flooding of the surfaces was not observed. The particular condensation behavior of each sample is discussed in the following.

**Figure 2:**
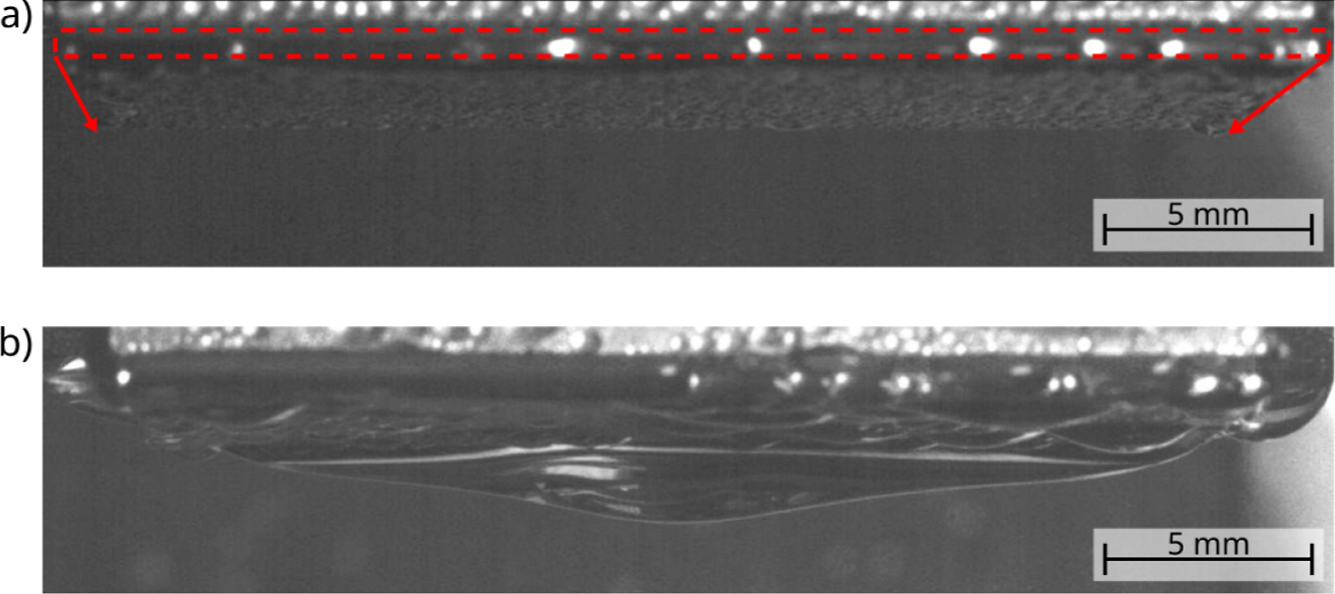
SV images of the Si sample after (a) 6 min and (b) 11.75 h of condensation. The red dashed box in (a) indicates the front face of the sample at the upper edge. The red arrows mark the sample length and extend to the bottom edge where the condensate accumulates with time forming large hanging droplets, as can be seen in (b).

In the case of the Si sample, a continuous growth of deformed droplets was observed across the hydrophilic surface by condensation and coalescence (Figure 2a). When their diameter is sufficiently large, they slide off under the action of gravity. However, the condensate is collected and pinned at the lower edge of the hydrophilic sample, forming hanging droplets of macroscopic dimensions. When their mass reaches a critical value, they partially detach. This process repeats continuously. After 11.75 h, the sample was still in DWC mode with hanging droplets of fluctuating size at its lower edge (Figure 2b).

For the F–Si sample, imaging showed that continuous droplet growth is followed by coalescence events. As the growing droplets approach a critical size, they slide off and clear the swept area of condensate. In contrast to the hydrophilic Si sample, no hanging water droplets were observed at the bottom of the hydrophobic region.

**Figure 3:**
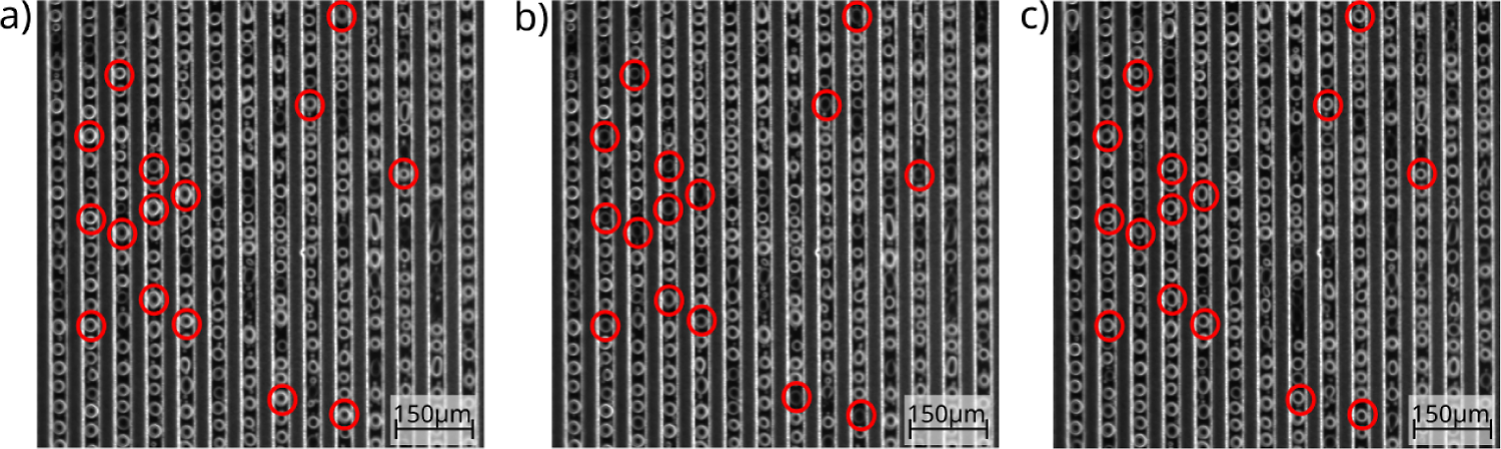
In the two consecutive surface images (a,b) (time delay 0.7 s) of the MSi-A sample, only droplets drained into the grooves were marked with a red circle. However, the image (b) shows that in most cases, the drainage was incomplete, leaving a lite contour behind with almost the same radius as the droplet removed. As can be seen in (c) [time delay 3.5 s with respect to image in (b)], the residual water at the marked locations in (b) acts again as condensation sites for droplet growth as condensation progresses with only small changes in radius.

The onset of condensation on the MSi-A sample is similar to that of the Si sample. Small droplets form on the top of the stripes as well as on the side and bottom walls of the grooves. The grooves are gradually filled by the continuous growth and coalescence of droplets inside the groove and by additional drainage of droplets from the stripe surface into the grooves, in accordance to the observations of Winter and McCarthy.^1^ The sequence of images in Figure 3a–c shows that this draining effect is incomplete since water stains remain on the stripes surface with a diameter equal to the width of the stripes. Newly formed droplets continue to grow until they come into contact with the water in the grooves and are sucked in by coalescence. Very rarely, larger droplets grow on the surface covering several stripes until they slide off due to the gravitational force and are pinned at the bottom edge of the sample. Drainage of the MSi-A surface is based on two sequential processes: the draining of the small droplets from the stripe surface into the grooves, followed by the drainage of this additional condensate via the grooves into the large hanging droplets at the bottom edge of the sample, driven by the Laplace pressure. The latter process is only observed if the hanging droplet is in contact with the water in the grooves.

**Figure 4:**
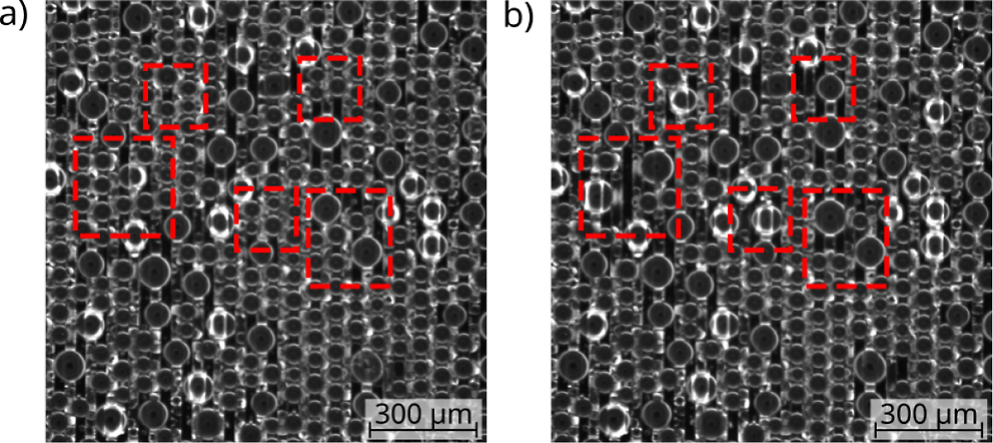
(a) Partially filled microstructure of a F-MSi-A sample before and (b) after coalescence. In the areas marked with red dashed boxes, the droplets inside and on top of the microstructure coalesce to form a larger droplet on top of the structure, continuously draining the groove droplets.

In the case of the F-MSi-A sample DWC occurred within and on top of the microgrooves, with a strong preference for the latter. In contrast to the MSi-A sample, the microgrooves were never completely flooded. Instead, the circular droplets on the striped surface grew to cover several stripes, while the droplets in the microgrooves disappeared after coalescing with the droplets on top of the stripes (Figure 4). Pinning of larger droplets at the lower sample edge occurred only occasionally.

**Figure 5:**
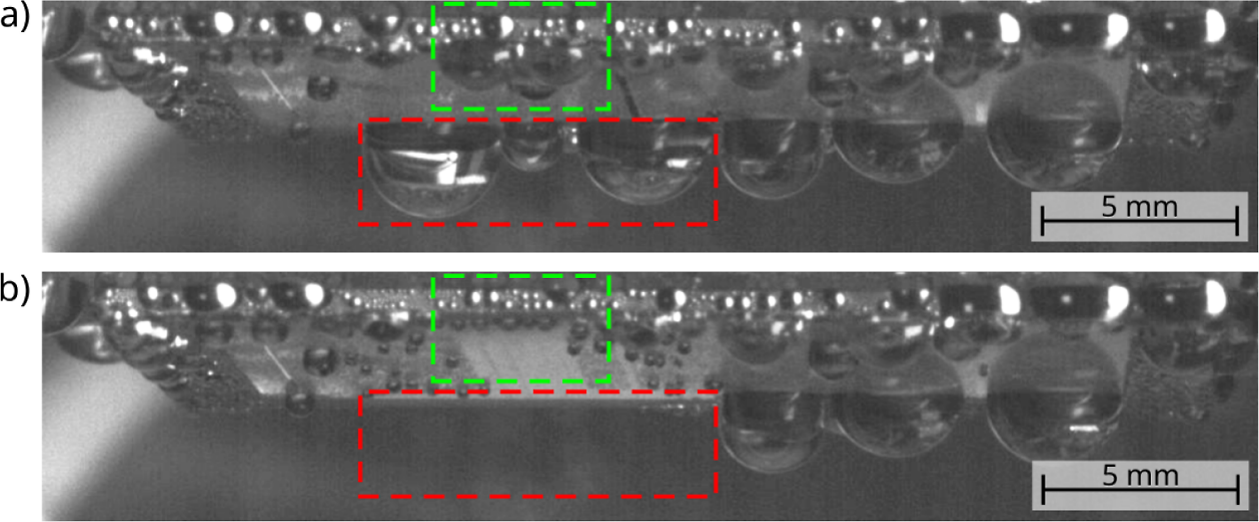
SV images of the PrMSi-A sample showing droplets with a diameter much larger than the groove width of W_0_ = 30 μm. (a) As the pinned droplets at the top (green dashed box) grew and slide off, they swept the surface and removed some droplets hanging at the lower edge (red dashed box). (b) After their removal, new smaller droplets formed spanning multiple grooves and stripes the swept droplets were in contact with. The same behavior can be observed for the PrMSi-I sample.

The biphilic PrMSi-I and PrMSi-A samples showed a very similar condensation behavior. DWC occurred both within the grooves and on top of the stripes. Over time, some of the top droplets disappeared, indicating droplet drainage into the microgrooves. The droplets on the walls of the grooves continued to grow and partially coalesced with each other, gradually filling up the groove with water. At the same time, the remaining droplets on the top of the stripes grew over several stripes. However, once the grooves were completely filled, a change in the condensation behavior was observed. The number of larger droplets growing over several stripes decreased as they were drained into the grooves. The remaining large droplets on the stripes slide off when their volume and therefore the gravitational force acting on them were sufficiently large. Only some of these droplets became pinned at the lower sample edge, forming hanging droplets with a large radius. The total number of clearly visible droplets on the stripes was low for those stripes and their adjacent grooves in contact with a hanging droplet. Due to the large radius of these hanging droplets and thus the low Laplace pressure drop across their menisci, these droplets suck up the excess water collected by the grooves from drainage of surface droplets (Figure 5a). Only after a hanging large droplet in contact with grooves drips off, larger droplets appeared again across multiple stripes, as can be seen in Figure 5.

In the case of the PrMSi-I and PrMSi-A samples, droplets were also formed at the same locations where drained droplets had shortly before disappeared. However, in contrast to the MSi-A sample here, the remaining wetted area left after a droplet drainage event is much smaller. The images show that the radius of a growing droplet on the stripes of the PrMSi-I and PrMSi-A samples increases continuously, in contrast to the balling-up behavior of the droplets starting growth at the water spots with their almost fixed area for the MSi-A sample. Additionally, we expected that droplets condensing near the edge of the stripes would maintain their position and thus coalesce with water in the groove at a smaller radius. However, most droplets continued to grow while simultaneously moving their center of mass toward the centerline of the stripe. These droplets were also not drained until they reached a diameter similar to the width W_0_ of the stripe.

### Discussion

In order to rationalize the unique results of the biphilic samples presented in the last section, we have implemented and adapted the HCT for image processing (Supporting Information, Section S3). It was used to analyze the droplet size distribution, the droplet volume, and the total number of droplets on the hydrophobic surface of the stripes only. We applied it to each of the 1010 images collected in the NV mode for both biphilic samples after 11.75 h of condensation when the grooves were already filled with water.

**Figure 6:**
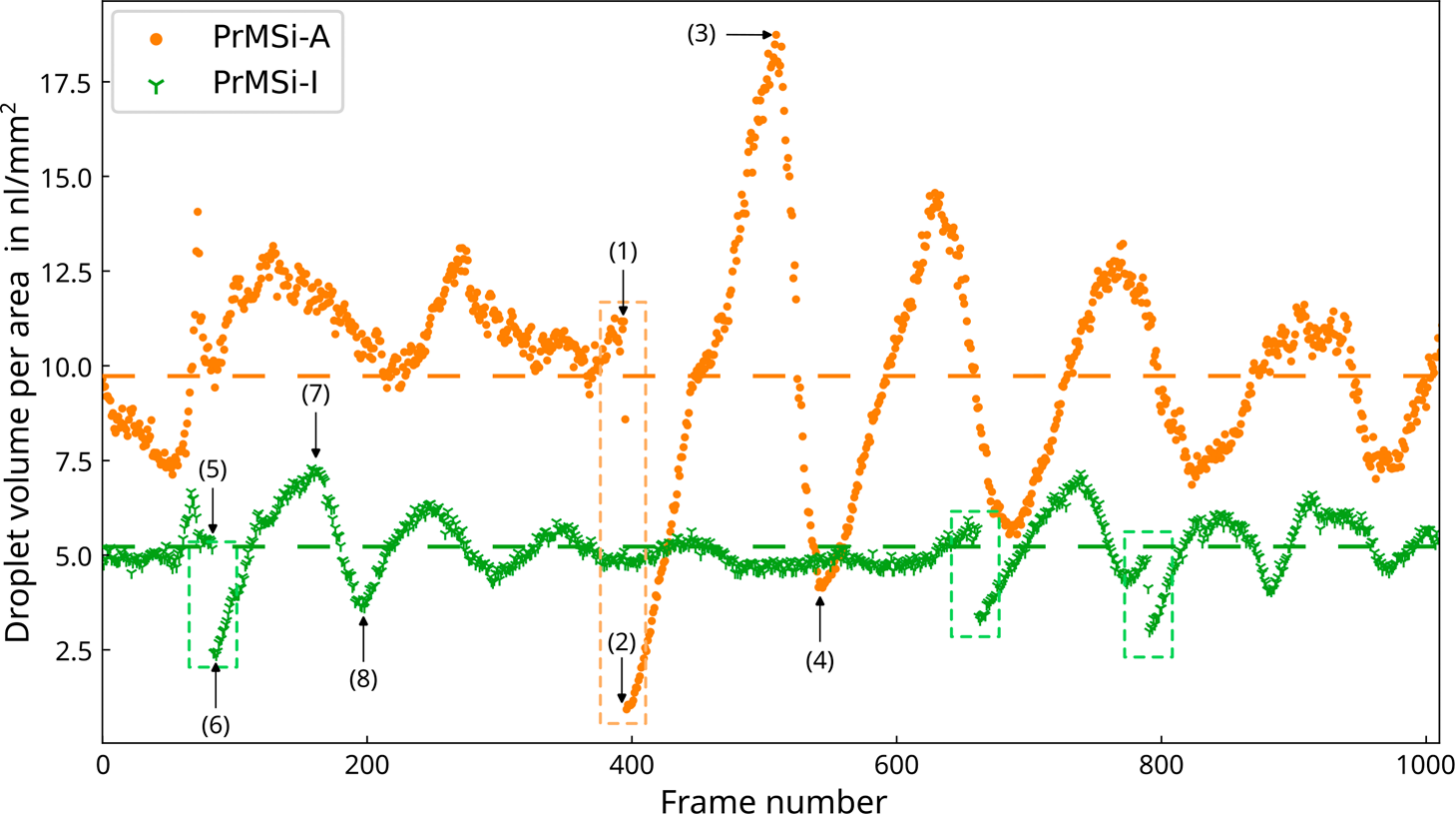
Calculated total droplet volume per area on the stripe’s surface for the PrMSi-A (orange) sample with an average of 9.9 nL/mm^2^ (orange dashed line) and the PrMSi-I (green) sample with an average of 5.2 nL/mm^2^ (green dashed line) for 1010 frames. The dashed boxes mark slide off events. Numbers have been added to indicate the condensation state before (1,5), after slide off (2,6), the first maximum (3,7) and the subsequent minimum (4,8) for the PrMSi-A and the PrMSi-I sample, respectively.

First, the total volume of water droplets on the hydrophobic stripes in the view field of the NV camera was calculated by taking the measured advancing angle of the water droplets on the photoresist material into account (Supporting Information, Section S1). It shows a nearly oscillatory time dependence with maxima indicating the highest condensate volume load on the stripes, followed by minima where most of the larger droplets were drained shortly before. Occasionally, this oscillatory behavior is disturbed when a larger droplet spanning multiple stripes slides off and clears almost all the stripes of water. These slide off events are marked with dashed boxes in Figure 6. In fact, these large droplets were not captured by the SV camera because they started outside the field of view and were much too fast for the chosen camera’s exposure time. After a slide off event, it takes a few oscillations with approximately exponential decaying amplitude before the stationary oscillation behavior of the water volume on the stripes surface is restored.

**Figure 7:**
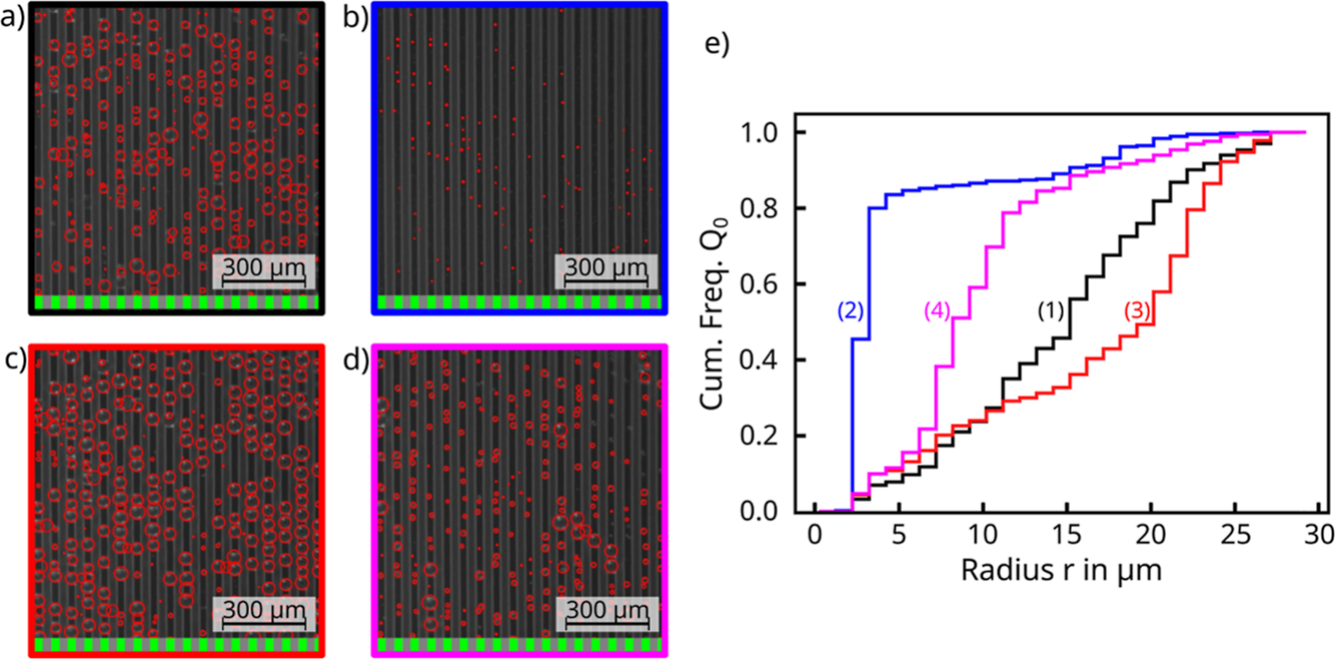
Analyzed images of the PrMSi-A sample obtained at the marked positions (1–4) in Figure 6: (a) before [black box, (1)], (b) after slide off [blue box, (2)], (c) at the first oscillation maximum where most droplets are close to their maximum radius and about to be drained [red box, (3)], and (d) at the subsequent oscillation minimum with most larger droplets drained [purple box, (4)]. The stripes with green and gray boxes below each image indicate the positions of the hydrophobic stripes and hydrophilic grooves, respectively. (e) Cumulative percentage of droplet radius for the images in (a–d). The line color of each graph corresponds to the box color of the corresponding image. The corresponding position numbers (1–4) from Figure 6 were added in (e) for clarity.

Because of the magnitude of the oscillation, it is instructive to examine the images at points (1) before, (2) immediately after slide off, (3) at the first maximum, and (4) the subsequent minimum for the PrMSi-A sample (Figure 7a–d). The cumulative droplet size distribution function (Figure 7e) of these four images reveals the significant variation of the droplet size distribution. The corresponding droplet size distributions can be found in the Supporting Information (Section S5).

Before the slide off event, the size distribution resembles a Gaussian function (Figure S4a bottom image, Supporting Information) and thus the course of the cumulative percentage [Figure 7e, graph (1)] is similar to the error function. Sweeping of the surface results almost in a step function behavior of the cumulative percentage due to the very narrow droplet size distribution of droplets with radius as small as 2–3 μm (Figure S4b bottom image, Supporting Information). These small droplets on the stripes (Figure 7b) and graph (2) in (e) act as condensation sites during the ongoing condensation. At the first maximum after slide off (Figure 7c) and graph (3) in (e), the cumulative percentage shows a strong increase of droplets with radius even larger than 20 μm. More than 60% of the droplets have a diameter larger than the stripe width of *W*_0_ = 30 μm and are already protruding from the stripe edges. Shortly afterward the water volume on the stripes reaches a minimum (Figure 7d) and graph (4) in (e) because most of the larger droplets were drained into the grooves. The rising edge of the cumulative percentage shifts to smaller values of the radius of 8–10 μm. Less than 20% of the droplets reach a diameter larger the stripe width *W*_0_ = 30 μm. If condensation continues without further slide off events, the oscillation amplitude of the cumulative percentage difference decreases. Otherwise the process just described will repeat itself.

**Figure 8:**
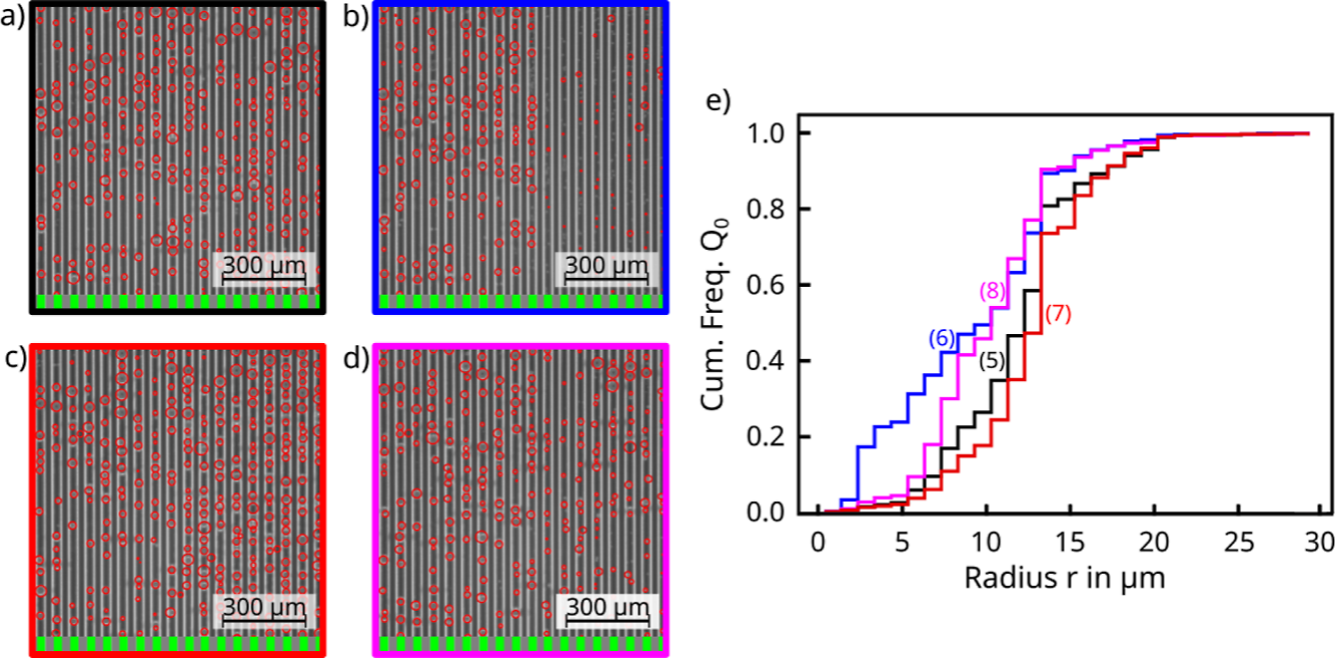
Analyzed images of the PrMSi-I sample obtained at the marked positions (5–8) in Figure 6: (a) before [black box, (5)], (b) after slide off [blue box, (6)], (c) at the first oscillation maximum when most droplets are close to their maximum radius and about to be drained [red box, (7)], and (d) at the subsequent oscillation minimum when most larger droplets have been drained [purple box, (8)]. The stripe with green and gray boxes below each image indicates the positions of the hydrophobic stripes and hydrophilic grooves, respectively. (e) Cumulative percentage of droplet radius for the images in (a–d). The line color of each graph corresponds to the box color of the corresponding image. The corresponding position numbers (5–8) from Figure 6 were added in (e) for clarity.

A similar behavior is observed for the PrMSi-I sample but with a much narrower cumulative percentage (Figure 8e). The corresponding droplet distribution can be found in the Supporting Information (Section S6). Here, at maximum 50% of the PrMSi-A sample is swept, resulting in a smaller amplitude of the oscillations and thus in smaller variations of the cumulative percentage. However, it can also be seen that more than 70% of the detected droplets have a radius < 15 μm for the analyzed images, while for the PrMSi-A sample at most 70% have a radius > 15 μm. This result indicates that droplets are drained earlier for the PrMSi-I samples, which is surprising since the width of the hydrophobic region should be identical for both samples. The thickness of the overhanging photoresist decreases due to the isotropic plasma etching process toward the free-hanging edge. This, along with a possible deformation of the stripes during processing, might explain the earlier drainage of droplets.

**Figure 9:**
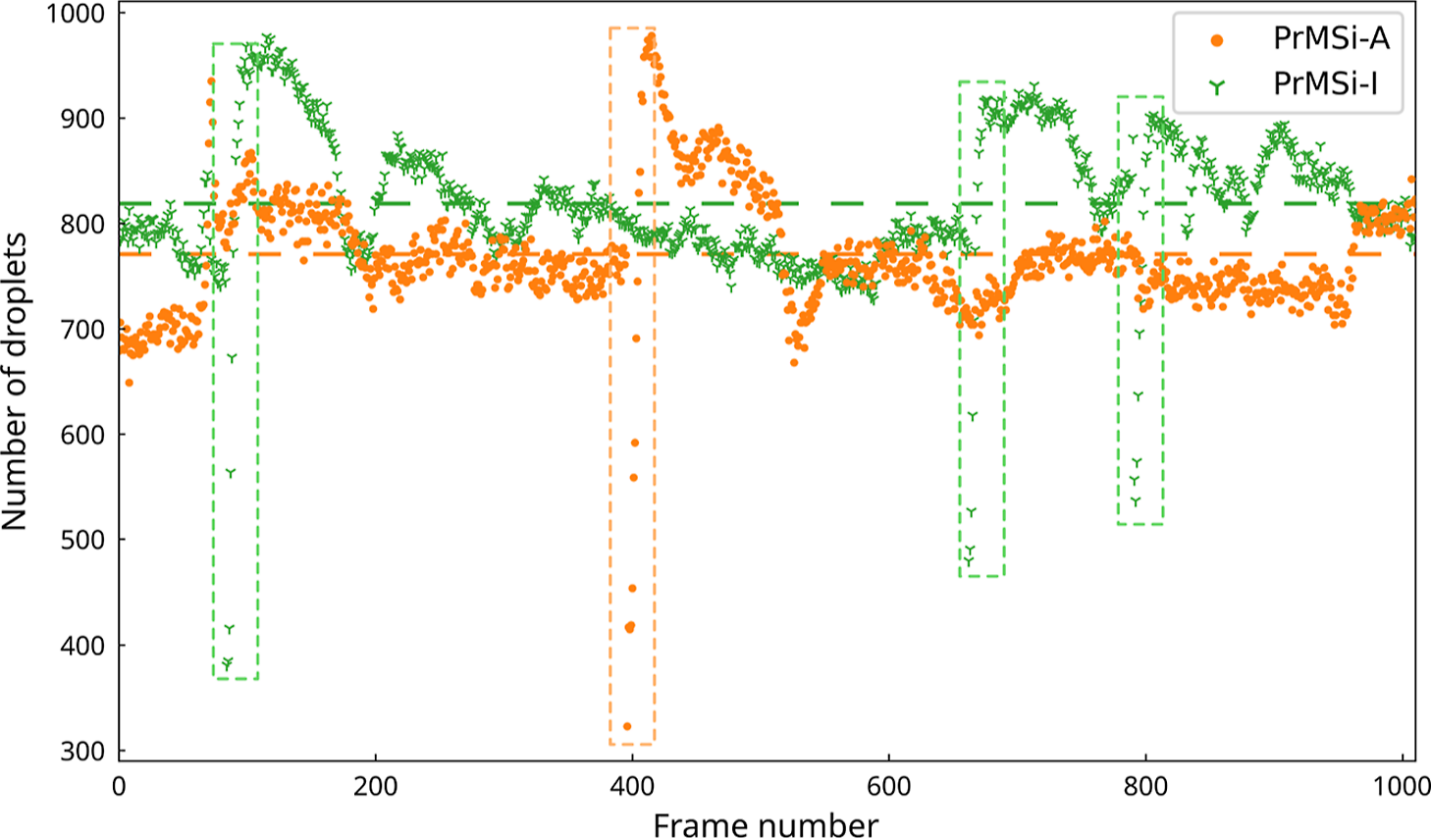
Number of droplets for the PrMSi-A sample (orange) with an average of 771 (orange dashed line) and for the PrMSi-I (green) with an average of 820 (green dashed line) as evaluated for the measurement data in Figure 6. The slide off events are marked with dashed light orange (PrMSi-A) and green (PrMSi-I) boxes.

Finally, it is interesting to note that the average number of droplets on the stripes of the PrMSi-I sample is 6% higher than on the PrMSi-A sample (Figure 9). However, the average droplet radius (Figure S6, Supporting Information) for the PrMSi-A sample (13.52 μm) is about 15% higher than that for the PrMSi-I sample (11.74 μm). Therefore, taking the droplet size distribution into account, the total evaluated droplet volume on the surface of the stripes on the PrMSi-A sample is larger by a factor of 1.9 than that on the PrMSi-I sample. However, the volume of water on the surface is the prerequisite for the process of water drainage via the grooves and therefore we observe a higher value of AoC for the PrMSi-A sample than for the PrMSi-I sample (Table 1).

## Conclusions

In this work we have shown that, by using microgrooved biphilic samples for condensation, the total amount of condensate can be increased by up to 15.9% (9.6%) compared to that of a flat hydrophilic (hydrophobic, i.e., carbon fluorocarbon coated) silicon surface. With ongoing condensation, first the grooves filled with water before droplet growth was observed on the hydrophobic stripe’s surface. The measured AoC shows the expected almost linear growth over time (Supporting Information, Section S4). However, local imaging during condensation followed by image processing with the adapted HCT algorithm for the biphilic samples revealed an oscillatory behavior of the overall water condensate volume on the hydrophobic surface of the photoresist stripes. The water volume was determined by evaluating the droplet size distribution of all images captured, assuming that each droplet forms a spherical cap with the measured advancing contact angle. We observed that condensate removal from the surface of the stripes was controlled by two processes: droplet drainage from the surface of the stripes into the microgrooves and the occasional slide off of larger surface droplets covering multiple stripes and grooves. During the slide off event of the larger droplets, the surface was cleared of water. Some of these droplets were pinned at the bottom edge of the sample, forming hanging droplets. When these were in contact with the grooves, they were continuously pumped up with the excess water of the grooves due to their Laplace pressure until they were large enough to drip off. Only then was it possible to form new droplets spanning multiple stripes and grooves.

## Supporting Information Available

Contact angle, experimental setup, droplet detection, total amount of condensate, droplet distribution for the PrMSi-A and PrMSi-I samples, and average droplet size (PDF)
