# Supplementary material for: Drainage during Condensation on Microgrooved Biphilic Surfaces
Source: Langmuir. 2023 Dec 29;40(2):1195–202. doi: 10.1021/acs.langmuir.3c02433 (PMC10795191; doi:10.1021/acs.langmuir.3c02433)
Supplement: Supplementary file 1 — la3c02433_si_001.pdf [file la3c02433_si_001.pdf]

Supporting Information:
Drainage during condensation on microgrooved biphilic surfaces

Additional information regarding contact angle, experimental setup, droplet detection, total amount of condensate, droplet distribution for the PrMSi-A and PrMSi-I sample and average droplet size.

Daniel Fotachov,[†] Raphael Raab,[‡] and Hans-Jörg Bart,[‡] Egbert Oesterschulze,[*,†]

†Department of Physics, Physics and Technology of Nanostructures, Rhineland-Palatinate Technical University of Kaiserslautern-Landau, Kaiserslautern 67663, Germany
‡Fluidverfahrenstechnik, Rhineland-Palatinate Technical University of Kaiserslautern-Landau, Kaiserslautern 67663, Germany

Table of Contents **Page**

Section 1:	Contact Angles	2
Section 2:	Experimental Setup	2
Section 3:	Droplet detection via Hough-Circle-Transform Algorithm (HCT)	3
Section 4:	Measured total amount of condensate (AoC)	4
Section 5:	Droplet distribution PrMSi-A	4
Section 6:	Droplet distribution PrMSi-I	5
Section 7:	Average droplet size	6

E-mail: oester@rptu.de

Section 1: Contact Angles

Contact angles were measured on flat surfaces using the needle-in method. A syringe is placed close to the surface and a drop is deposited. This drop is inflated to 40 µl and then deflated to 0 µl while the contact angle is measured. This process is repeated three times on each sample at different locations. During inflation the advancing contact angle and during deflation the receding contact angle are measured, respectively. The advancing contact angle was used as an upper limit for the contact angle to calculate the droplet volume on the stripe's surface for each image frame. The contact angle was measured for an etched flat silicon (Si), a flat photoresist coated surface (PrSi) and the flat fluorocarbon-coated surface (F-Si).

Measured advancing contact angle θ_{CA}:

Si: 87°
PrSi: 120°
F-Si: 128°

The volume of a water droplet with advancing contact angle θ_{CA} was evaluated as:

$$V = \frac{\pi}{3} r^3 (4 - (2 + \cos\theta_{CA}) \cdot (1 - \cos\theta_{CA})^2)$$

assuming that it forms a spherical cap. Due to the small size (radius < 20 µm), the impact of gravity on the droplet shape can be neglected.

Section 2: Experimental Setup

A schematic and an image of the experimental setup are shown in Figure S1.

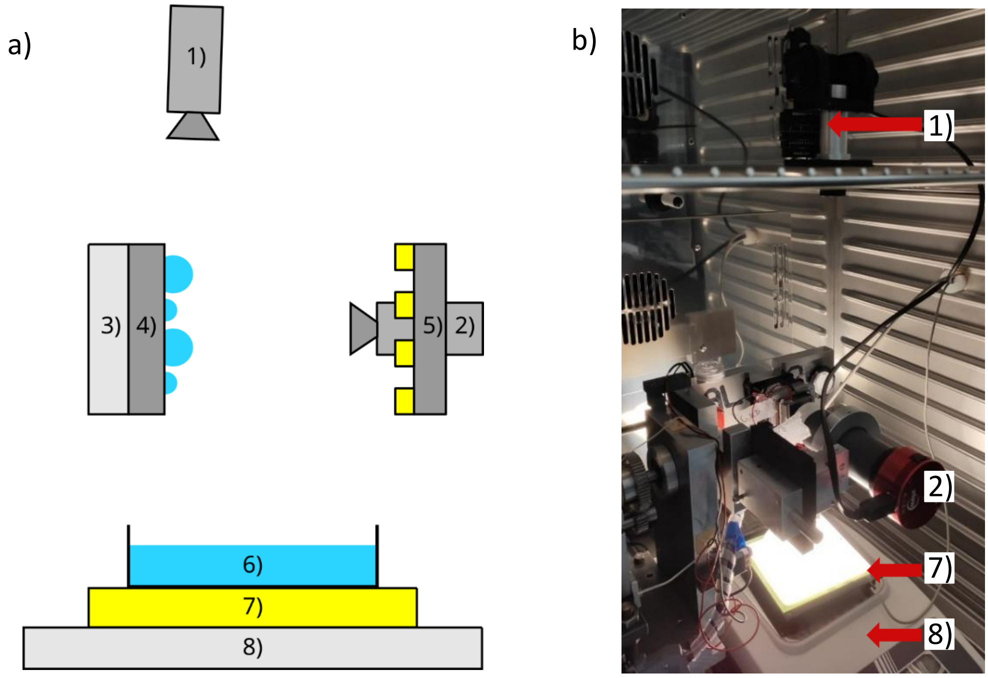

Figure S1: a) Scheme of the experimental setup consisting of: cameras for sample imaging at 1) grazing incidence (SV) and 2) normal to the sample surface (NV), 3) sample chiller, 4) sample with condensate, 5) ring LED, 6) Petri dish with captured condensate, 7) OLED screen, 8) scale. b) Optical image of the experimental setup inside the climate chamber while the goniometer is inclined by 90°.

Two different cameras were used for imaging during the condensation. One camera was installed at a viewing angle of about 5° to the surface (1) IDS UI-336xCP-M (2048x1088 pixels) and another perpendicular to the surface (2) ASI 1600 MM Mono, 4656x3520 pixel). The first camera was used to image the condensation and dropelt slide off behavior on a macroscopic scale (SV mode). To keep the frame rate of 60 fps constant for the given illumination intensity, only 1072x300 pixels of the full camera chip were recorded. A 10x magnification lens was mounted on the second perpendicular camera, to observe the microscopic behavior (NV mode). NV-images of the sample were taken every 0.7 seconds with an exposure time of 0.5 seconds. The sample (4)) was attached with thermally conducting adhesive tape to the sample cooler (3) UEPT-KIT3-Pt100), which consisted of a fan (UEPK-Lu-12-60), a Peltier cooling element (UEPT-140-127-040C200S) and an aluminum cube used as a sample holder. The dripped off condensate was collected with a Petri dish (6)) placed on an OLED screen (7)) mounted on a scale (8) Kern EWJ 300-3). This allowed the total amount of condensate (AoC) to be measured over the course of the experiment. Illumination of the sample during imaging was done with an OLED screen for SV mode and an LED ring light for NV mode. The sample cooler (3)), the NV camera (2)) and the ring LED (5)) were placed on a goniometer that can be tilted by up to 90°. To ensure stable boundary conditions (relative humidity: 60%, chamber temperature: 30°C), the entire experiment was conducted inside an environmental chamber (Memmert HPP 260). The cooling temperature of the sample cooler was 6°C and was controlled throughout the experiment by a built-in PT100 temperature sensor.

Section 3: Droplet detection via Hough Circle Transform algorithm (HCT)

To increase the number of successfully detected droplets, certain adjustments were necessary. First, by subtracting a frame with no condensate on the frame being studied, reflections from the surface itself are almost removed (Fig. S2 a) - b)). Instead of using only one set of parameters for the HCT, different sets of parameters should be used for specific droplet ranges. The first set of parameters should include the largest total radius from which the studied radius decreases (Fig. S2 c)). In our work, we used 3 different sets of parameters, with specific parameters for each radius class. These parameters are highly dependent on illumination and data type. To ensure that droplets are not detected multiple times, they were replaced by black dots after their detection (Fig. S2 d)). To correctly detect smaller droplets, it can be helpful to cut off gray values that do not correspond to the droplets. The complete evaluation of a single image takes approx. 4 seconds.

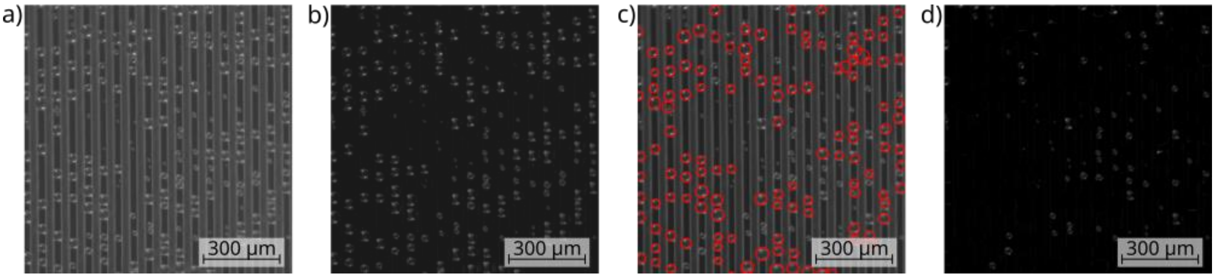

Figure S2: a) original image, b) image after background subtraction, c) analyzed droplets with specific radii marked with a red circle, d) blocked areas inside the analyzed image.

Section 4: Measured total amount of condensate (AoC)

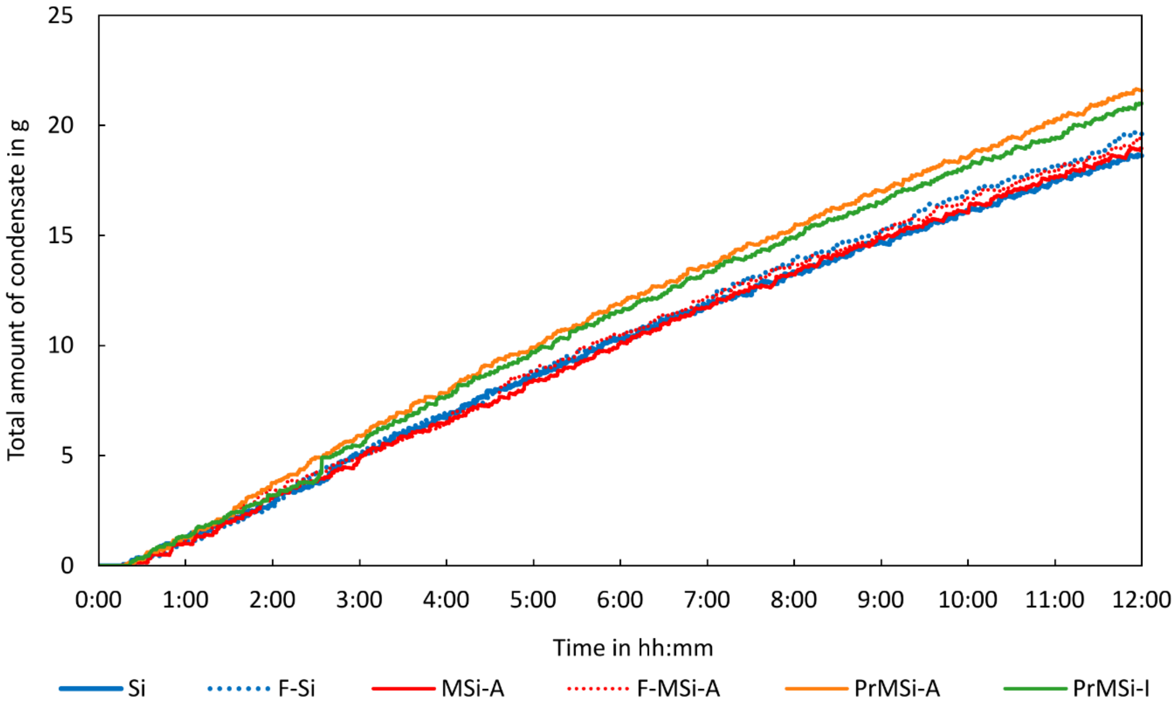

Figure S3: Measured total amount of captured condensate over a 12 hours period for each sample. The maximum values of AoC are obtained for the biphilic samples (PrMSi-A and PrMSi-I).

Figure S3 shows the total amount of condensate (AoC) captured over a 12 hour time period for each investigated sample. In order to be able to operate both cameras close to the cooled sample, we had to set up a Petri dish of larger diameter at a relatively large distance from the sample to collect all droplets falling from the lower sample edge. However, the Petri dish filled only slowly, resulting in a continuously increasing water/air surface area. The latter is prone to enhanced evaporation if we take the also small flow rate of the injected humid air stream and of the fan of the Peltier sample cooler into account. This may explain the observed non-linear time dependence of the collected condensate mass. The biphilic samples show the largest AoC over the course of the experiment, with a maximum of 21.59 g and 20.99 g for the PrMSi-A for PrMSi-I sample, respectively.

Section 5: Droplet distribution PrMSi-A

Figure S4 shows the droplet distribution of the sample PrMSi-A sample before a) and immediately after b) a slide off event. This is followed by a decreasing oscillation with its corresponding first maximum c) and subsequent minimum d). Before slide off we have a broad bell-shaped droplet distribution centered at 15 µm radius and extending to almost the width of the stripes (30 µm) (Fig. S4 a)). This distribution changes dramatically after slide off to a peaked and narrow curve centered at 3 µm (Fig. S4 b)). However, the number of small droplets (3 – 4 µm) exceeds that from the distribution for a) by a factor of 6.88 and 4.67, respectively. This shows that the slide off event is incomplete, leaving many small droplets on the stripe's surface. They subsequently act as condensation sites for the next generation of droplets during the ongoing condensation process. When the total water volume reaches

its maximum value two bell shaped distribution centered at 6 µm and 20 µm are superimposed (Fig. S3 c)). At this point most of the larger droplets exceed the rim of the stripes and are cleared from the stripe's surface by coalescence with the water in the grooves. Therefore, only the bell-shaped distribution of about 7 - 8 µm mean radius is left on the surface (Fig. S3 d)).

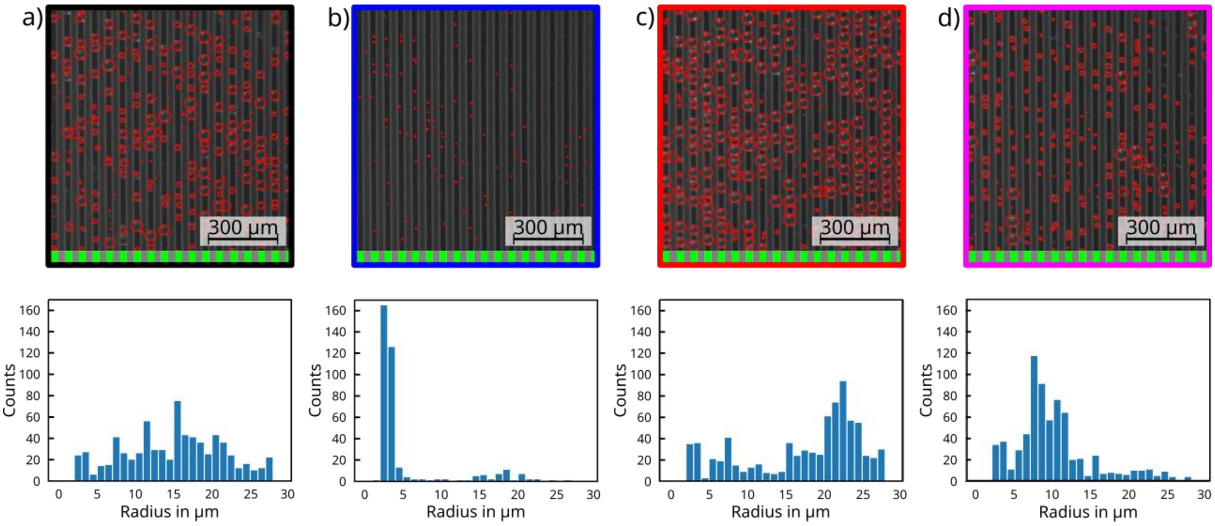

Figure S4: Analyzed images and their corresponding droplet distribution of the PrMSi-A sample at the marked position (1) – (4) in Fig. 6: a) before (black box, (1)), b) after slide off (blue box, (2)), c) at the first maximum where most droplets close to their maximum radius and about to be drained (red box, (3)) and d) the subsequent minimum with most bigger droplets drained (purple box, (4)). Green (gray) boxes at the lower side of the image represent the locations of the hydrophobic stripes (hydrophilic grooves).

Section 6: Droplet distribution PrMSi-I

Figure S5 shows the droplet distribution of the PrMSi-I sample before (a)) and immediately after (b)) a slide off event. This is followed by an oscillation of decreasing amplitude with its corresponding first maximum (c)) and subsequent minimum (d)). Before slide off, we have a broad bell-shaped droplet distribution with a peak at 14 µm radius extending to 22 µm (Fig. S5 a)). After about 50% of the surface is swept, this peak is greatly reduced, while the number of droplets with radius < 5 µm increases (Fig. S5 b)). This shows that the slide off event is incomplete, leaving many small droplets on the strip surface. They subsequently act as condensation sites for the next generation of droplets in the ongoing condensation process. Due to the similar condensation rate of the remaining droplets, the total water volume reaches its maximum value. Here, the number of droplets with a radius of 14 µm exceeds 240 counts (Fig. S5 c)). At this point, the larger droplets protrude the edges of the strip and are removed from the strip surface by coalescing with the water in the grooves (Fig. S5 d)). Comparing the droplet distribution of the PrMSi-A sample with that of the PrMSi-I sample reveals that droplets on the latter have a smaller radius before they are cleared. We assume that the overhanging photoresist was slightly thinned by the plasma etching process and presumably bends down a little due to the droplets mass and its own weight, which leads to earlier contact between the droplets and the filled grooves and therefore faster draining.

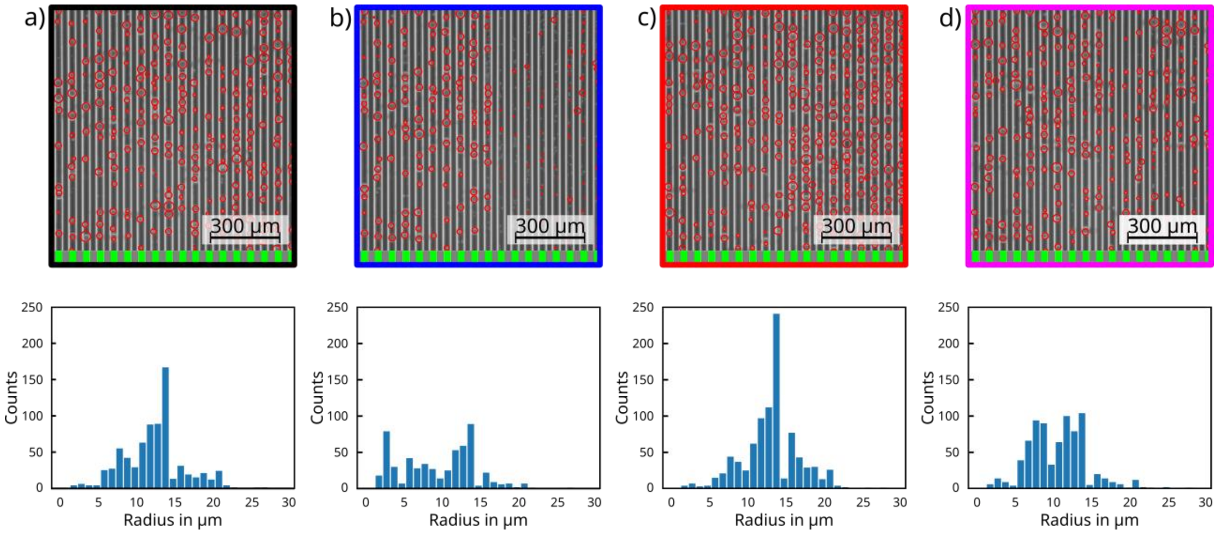

Figure S5: Analyzed images and their corresponding droplet distribution of the PrMSi-I sample at the marked position (5) – (8) in Fig. 6 (see manuscript): a) before (black box, (5)), b) after slide off (blue box, (6)), c) at the first maximum where most droplets are close to their maximum radius and about to be drained (red box, (7)), and d) the subsequent minimum with most bigger droplets drained (purple box, (8)). Green (gray) boxes at the lower side of the image represent the locations of the hydrophobic stripes (hydrophilic grooves).

Section 7: Average droplet size

Transient average droplet radius distribution for the biphilic samples for each frame starting after 11.75 hours of condensation. The overall averaged radius of droplets for the PrMSi-A sample is 15% higher (13.5 µm) than for the PrMSi-I sample (11.7 µm).

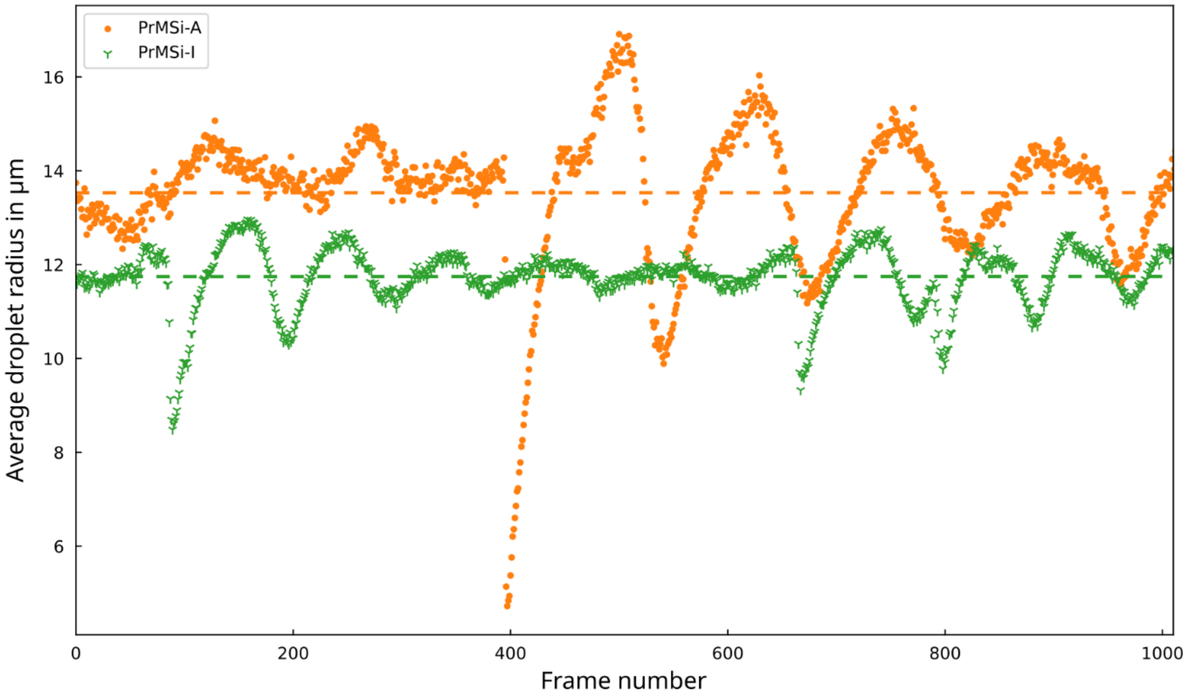

Figure S6: Transient average droplet radius for each frame for PrMSi-A (orange) and PrMSi-I (green) with an overall average radius of 13.5 µm (orange dashed line) and 11.7 µm (green dashed line) for the PrMSi-A and PrMSi-I sample, respectively.
